# Malignant mesothelioma of the tunica vaginalis testis initially misdiagnosed as a benign testicular hydrocele: a case report

**DOI:** 10.3389/fonc.2026.1902555

**Published:** 2026-09-08

**Authors:** Chaohui Zhang, Hang Su, Liuxiong Guo, Zehua Li, Jue Wang, Junbo Chen, Gang Wang

**Affiliations:** 1 Department of Graduate School, Hebei Medical University, Shijiazhuang, Hebei, China; 2 Department of Urology, Hebei General Hospital, Shijiazhuang, Hebei, China

**Keywords:** diagnosis, malignant mesothelioma, prognosis, testicular hydrocele, treatment, tunica vaginalis testis

## Abstract

**Objective:**

To improve the clinical understanding, diagnostic accuracy, and therapeutic management of mesothelioma of the tunica vaginalis testis.

**Methods:**

We present a case of mesothelioma of the tunica vaginalis in a patient recently admitted to our institution. The clinical characteristics, histopathological features, and therapeutic modalities used are described. FAPI PET/CT was applied as an adjunctive diagnostic tool for preoperative surgical planning. A narrative review of the relevant literature was conducted to discuss current diagnostic approaches, treatment options, and prognostic outcomes.

**Results:**

Postoperative histopathological examination revealed an epithelioid mesothelioma of the left testis. The tumor infiltrated the visceral tunica vaginalis and extended outward into the subcutaneous fibrous connective tissue of the scrotum. The infiltration also extended through the parietal tunica vaginalis to the tunica albuginea and involved the smooth muscle of the spermatic cord and the pampiniform venous plexus, with formation of a tunica vaginalis cyst. The adjacent testicular parenchyma and epididymis were not involved, and the spermatic cord surgical margin was free of malignancy.

**Conclusion:**

Mesothelioma of the tunica vaginalis is a rare malignancy with distinctive histopathological features. Because its clinical presentation resembles that of hydrocele, it is readily misdiagnosed. This case underscores the need to maintain a high index of suspicion when evaluating patients who present with hydrocele. The absence of recurrence at the limited 2-month follow-up does not permit any conclusion regarding disease control, cure, or recurrence, and prolonged surveillance is required. The efficacy and necessity of postoperative adjuvant treatment—including chemotherapy, radiotherapy, and targeted therapy—warrant further validation in prospective studies.

## 1 Introduction

Malignant mesothelioma (MM) is a rare neoplasm originating from the mesothelial cells that line serosal surfaces, and it can involve the pleura, peritoneum, pericardium, and the tunica vaginalis testis. Primary involvement of the tunica vaginalis is exceedingly uncommon (1), accounting for less than 1% of all reported MM cases (2). Because of this rarity, established treatment regimens are largely derived from case reports, small case series, and retrospective reviews. Here, we present a recent case of tunica vaginalis mesothelioma managed at our institution. By combining this case with the current literature, we aim to provide a detailed clinical analysis and to examine the main challenges in its diagnosis, treatment, and prognostic evaluation.

## 2 Case presentation

**Figure 1:**
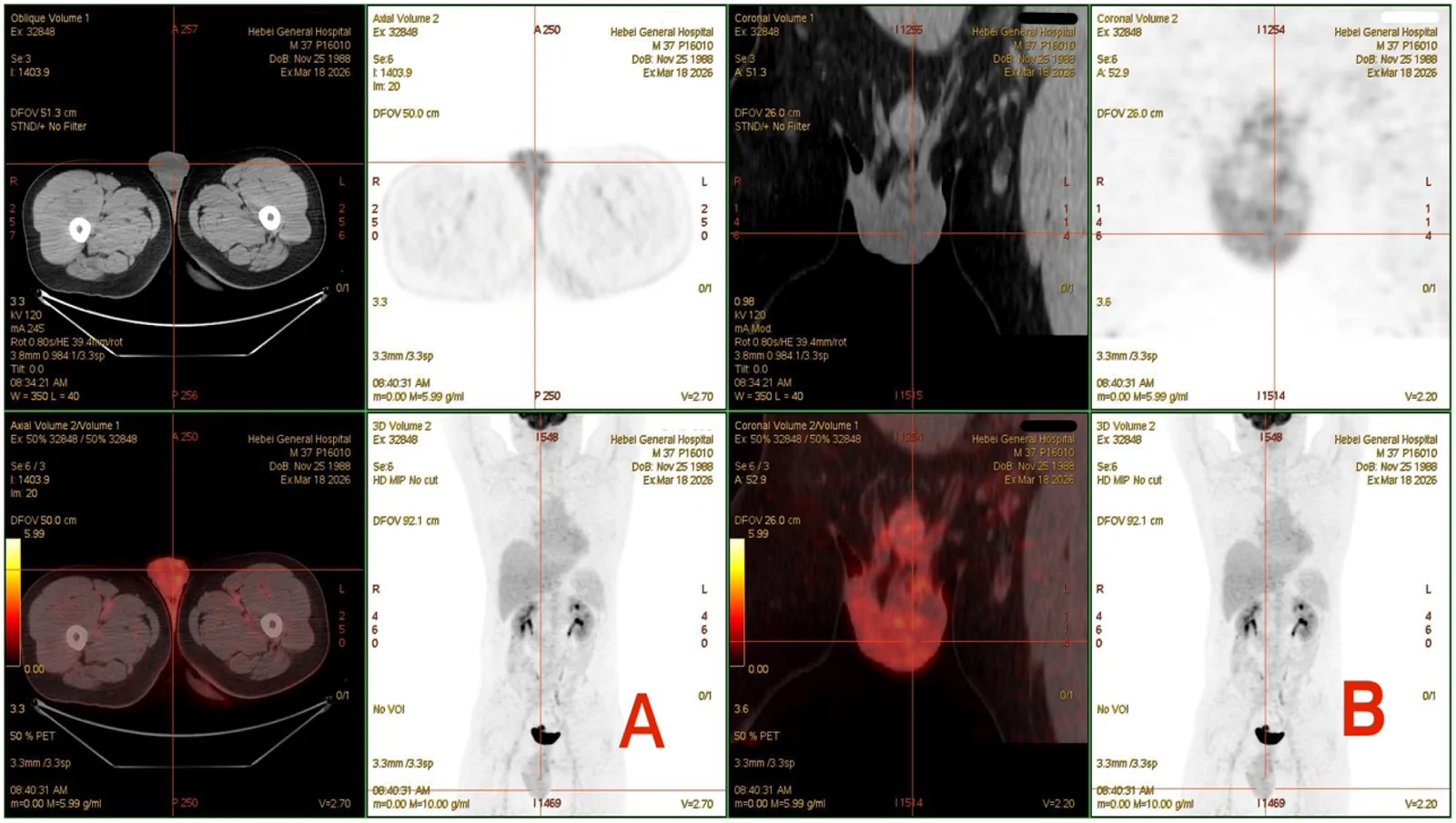
Positron emission tomography/computed tomography (PET/CT) imaging of the scrotal lesion. **(A)** Axial views encompassing unenhanced CT (upper left), PET (upper right), fused PET/CT (lower left), and a whole-body maximum intensity projection (MIP) image (lower right). The cross-sectional images clearly demonstrate an enlargement of the left hemiscrotum characterized by increased internal soft tissue density when compared to the normal contralateral side. This structurally abnormal region exhibits mild metabolic activity on the corresponding PET and fused images. **(B)** Coronal views displaying unenhanced CT (upper left), PET (upper right), fused PET/CT (lower left), alongside the whole-body MIP image (lower right). These longitudinal sections further characterize the craniocaudal extent and morphological boundaries of the left scrotal soft tissue lesion, confirming the localized mild metabolic uptake.

**Figure 2:**
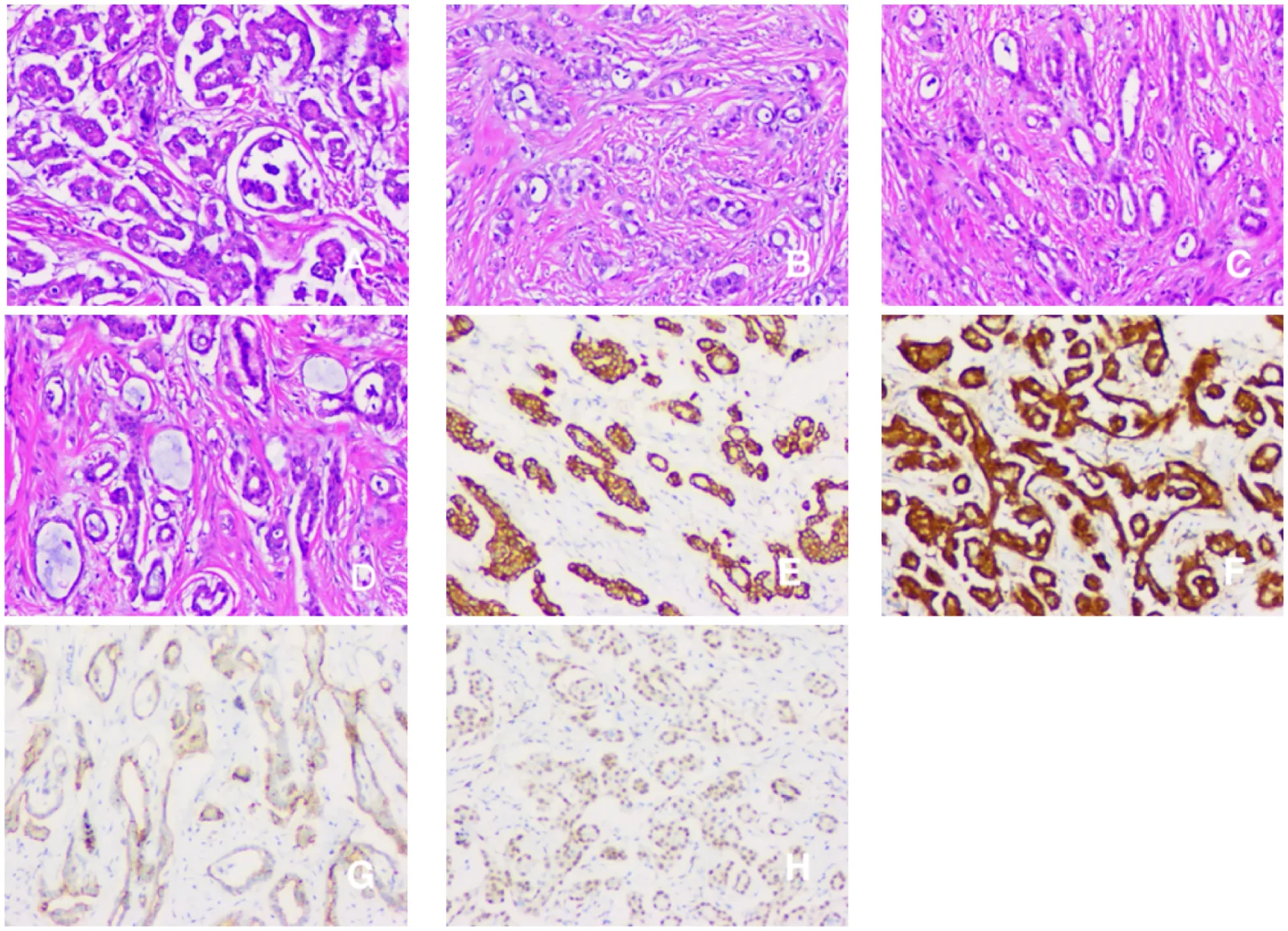
**(A)** The neoplastic tissue is arranged in a micropapillary architectural pattern, comprising cuboidal to polygonal tumor cells endowed with abundant and distinctly eosinophilic cytoplasm (Hematoxylin and eosin stain, original magnification ×100). **(B)** The tumor exhibits glandular and irregular slit-like structural formations, which are accompanied by a pronounced proliferation of the interstitial fibrous stroma (Hematoxylin and eosin stain, original magnification ×100). **(C)** The neoplastic cells display conspicuous cytological atypia, characterized by irregular nuclear contours and the presence of hyperchromatic, prominent nucleoli (Hematoxylin and eosin stain, original magnification ×100). **(D)** The neoplastic tissue predominantly exhibits a glandular and microcystic growth pattern (Hematoxylin and eosin stain, original magnification ×100 **(E)** Immunohistochemical analysis reveals positive expression of CKpan within the neoplastic tissue (Original magnification ×100). **(F)** Immunohistochemical analysis reveals positive expression of CR within the neoplastic tissue (Original magnification ×100). **(G)** Immunohistochemical analysis reveals positive expression of D2–40 within the neoplastic tissue (Original magnification ×100). **(H)** Immunohistochemical analysis reveals positive expression of WT-1 within the neoplastic tissue (Original magnification ×100).

**Figure 3:**
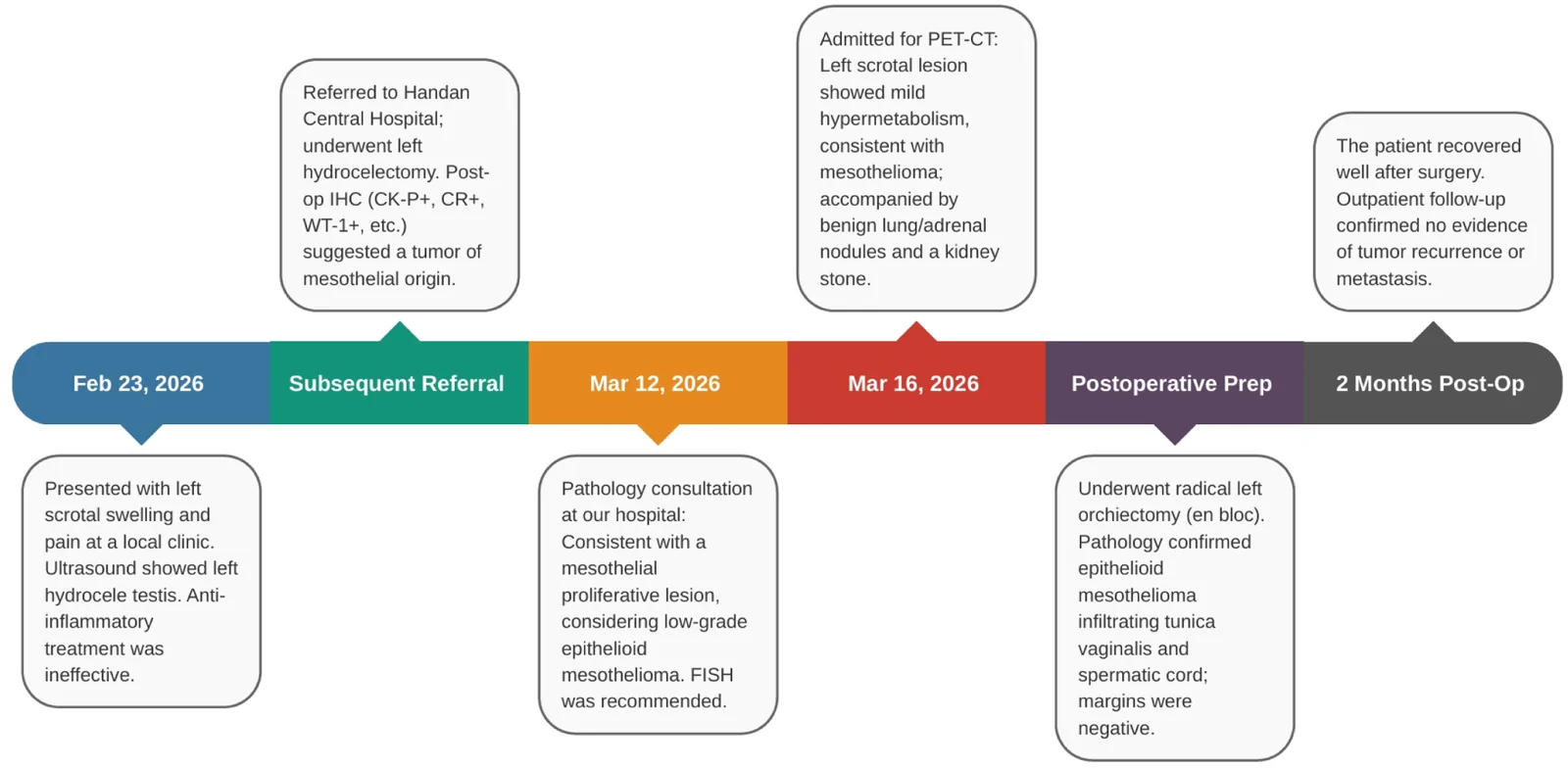
Treatment timeline of this case.

A 37-year-old man was admitted to our institution with a four-day history of left scrotal swelling. The patient first noted painful swelling in the left scrotum on February 23, 2026. Scrotal color Doppler ultrasound at a local clinic suggested a “left testicular hydrocele.” His symptoms did not improve after symptomatic anti-inflammatory therapy. For further evaluation, he presented to Handan Central Hospital, where a repeat scrotal ultrasound confirmed the left testicular hydrocele and a left hydrocelectomy (tunica vaginalis eversion) was performed. Intraoperatively, turbid yellowish fluid was drained. After symptomatic postoperative care, the patient recovered and was discharged. Immunohistochemical (IHC) staining performed at the referring hospital showed the following profile: CK-P (+), CD30 (-), CR (+), MOC-31 (-), P53 (no abnormal expression), MC (+), EMA (weak +), WT-1 (+), BAP1 (nuclear +), anti-Inhibin (-), Ber-EP4 (-), Desmin (-), and a Ki-67 proliferation index of approximately 10%. The pathological specimens were then sent to our hospital for expert consultation. On review of the initial IHC findings, our Pathology Department concluded that the tunica vaginalis lesion was consistent with a mesothelial proliferative disorder, highly suggestive of a low-grade epithelioid mesothelioma. Fluorescence *in situ* hybridization (FISH) for CDKN2A alterations was recommended to confirm the diagnosis. For definitive management, the patient was admitted to our institution on March 16, 2026. PET/CT revealed the following findings (**Figure 1**): (1) A ^68^Ga-FAPI PET/CT scan showed increased soft-tissue density in the left scrotum (approximately 40 × 38 × 31 mm) with mild, heterogeneous FAPI uptake (SUV max = 3.2). No FAPI-avid retroperitoneal or inguinal lymphadenopathy and no distant metastatic lesions were identified. The scan had been obtained approximately three weeks after the initial hydrocelectomy. Therefore, FAPI imaging alone was insufficient to definitively characterize the scrotal lesion; however, when integrated with the pathological findings, mesothelioma was considered the most likely diagnosis. (2) Multiple non-metabolic micronodules in the right middle and left lower pulmonary lobes, presumed benign and recommended for follow-up; concurrent hepatic steatosis; a non-metabolic, low-density nodule in the body of the left adrenal gland, suspected to be an adenoma and requiring observation; and left renal calculi. After preoperative preparation, a radical left orchiectomy was performed. An oblique 6 cm incision was made in the left groin to expose the spermatic cord, which was mobilized proximally to the internal inguinal ring. The vas deferens and spermatic vessels were isolated, doubly ligated, and transected. The left testis was then delivered through the incision. Extravaginal dissection was performed, followed by division of the gubernaculum testis, allowing en bloc removal of the specimen (approximately 35 mm × 25 mm × 20 mm). After hemostasis was achieved, a Penrose drain was placed through a dependent stab incision at the base of the scrotum, and the incision was closed in layers. Postoperative pathological evaluation confirmed an epithelioid mesothelioma of the left testis. The tumor infiltrated the visceral tunica vaginalis and extended outward into the subscrotal fibrous connective tissue. It also invaded the parietal tunica vaginalis down to the tunica albuginea and infiltrated the smooth muscle of the spermatic cord and the pampiniform plexus, with formation of a hydrocele sac. The testicular parenchyma and epididymis were not involved by tumor, and the spermatic cord margins were tumor-free. The pathology report lacked documentation regarding the specific distance to the negative margin. The diagnosis of malignancy was based primarily on this unequivocal invasive growth into the tunica albuginea, spermatic cord smooth muscle, and pampiniform plexus, together with cytological atypia and a confirmatory mesothelial immunophenotype (3). Retained nuclear BAP1 expression did not exclude malignancy (4); CDKN2A/p16 FISH could not be performed because the patient and his family declined the recommended testing for financial reasons. The final IHC panel showed (**Figure 2**): CK pan (+), Vimentin (+), CR (+), D2-40 (+), WT-1 (+), Desmin (-), Glut-1 (+), EMA (+), and a Ki-67 index of approximately 5%. Primary malignant mesothelioma of the tunica vaginalis testis is an exceedingly rare malignancy for which no widely accepted, dedicated TNM staging or grading system has been established (5). Given that staging schemes devised for other testicular tumors may not accurately reflect the biological behavior of mesothelioma, we preferred to describe the anatomical extent of disease on the basis of histopathological findings. In the present case, the tumor infiltrated the visceral tunica vaginalis and extended outward into the subcutaneous fibroconnective tissue of the scrotum. It penetrated the parietal tunica vaginalis to involve the tunica albuginea and infiltrated the smooth muscle of the spermatic cord and the pampiniform plexus. Notably, the testicular parenchyma and epididymis were uninvolved, and the spermatic cord surgical margin was free of tumor. These features are indicative of locally advanced disease, with tumor extension beyond the tunica vaginalis to adjacent structures but without involvement of the testis proper or any documented metastatic dissemination. At the 2-month outpatient follow-up, there was no clinical or radiological evidence of recurrence (**Figure 3**). This interval is, however, too short to assess oncological control, and the patient remains under active long-term surveillance.

## 3 Discussion

Since the first report of this entity by Barbera and Rubino in 1957, fewer than 300 cases have been reported worldwide to date (5). The epidemiology and exact risk factors of malignant mesothelioma of the tunica vaginalis testis (MMTVT) remain poorly understood. Although asbestos exposure is widely recognized as a primary cause of MM (6), a retrospective review of 223 cases by Bisceglia et al. (7) found that only 30% to 40% of these patients had a documented history of asbestos exposure.

### 3.1 Clinical characteristics of MMTVT

Because its clinical presentation is nonspecific, this malignancy usually presents as a painless testicular mass, scrotal swelling, or hydrocele, and is occasionally accompanied by orchitis or epididymitis (8). In our case, the tumor was initially misdiagnosed as a benign testicular hydrocele. The absence of pathognomonic radiological signs on computed tomography (CT) or magnetic resonance imaging (MRI) further increases the risk of misdiagnosis (9); a definitive diagnosis therefore depends mainly on histopathological and immunohistochemical (IHC) evaluation.

### 3.2 Histopathological features of MMTVT

Mesothelioma of the tunica vaginalis testis originates from the mesothelial cells lining the inner aspect of the scrotum and the external surface of the tunica albuginea (10). Histologically, it is broadly classified into three primary subtypes: epithelioid, sarcomatoid, and biphasic (11). The epithelioid subtype is the most common (approximately 60% of cases), followed by the biphasic (22%) and sarcomatoid (approximately 1%) variants. A study by Drevinskaite et al. (12) found that the biphasic subtype is associated with significantly worse survival. A definitive diagnosis of MMTVT requires histological and IHC examination of orchiectomy specimens. According to the 2023 consensus guidelines of the International Mesothelioma Interest Group (IMIG), IHC assessment should be based on a combinatorial panel comprising at least two positive mesothelial markers and at least two epithelial (carcinoma) markers, the latter serving to exclude metastatic or paratesticular carcinoma (13, 14). Commonly used positive mesothelial markers include calretinin, cytokeratin 5/6, WT-1, and D2-40, whereas epithelial markers include MOC-31, Ber-EP4, carcinoembryonic antigen (CEA), and claudin-4. Among the mesothelial markers, calretinin is one of the most reliable for distinguishing mesothelioma from adenocarcinoma; however, it is not entirely specific, as focal calretinin expression has been reported in certain adenocarcinomas, including pancreatic and pulmonary carcinomas (14). Consequently, no single marker is diagnostic, and the diagnosis must rest on integrated evaluation of the overall immunophenotype and morphology. In the present case, the mesothelial immunophenotype (pan-CK+, calretinin+, D2-40+, WT-1+, EMA+), together with negative epithelial markers (MOC-31−, Ber-EP4−) and, in particular, the presence of unequivocal destructive stromal invasion, established the diagnosis of epithelioid mesothelioma.

### 3.3 Differential diagnosis for MMTVT

The diagnosis of MMTVT requires careful exclusion of several potential mimics. Reactive mesothelial hyperplasia is a well-recognized diagnostic pitfall of the tunica vaginalis, but it lacks the true stromal invasion and deep infiltrative features required to define malignancy (3). Adenomatoid tumor is the most common mesothelial-derived neoplasm of the paratesticular region; it is benign and well-circumscribed and shows gland-like spaces of variable pattern, ranging from almost solid cords of cuboidal and low-columnar cells to greatly dilated spaces lined by markedly flattened cells, without destructive invasion or cytological atypia (15).Well-differentiated papillary mesothelioma is characterized by a single layer of cells covering fibrovascular papillary cores, with bland cytomorphology and no or only superficial invasion; this contrasts with the cytological atypia and the prominent infiltrative microcystic/glandular growth pattern observed in the present case (16). Metastatic carcinoma and paratesticular serous tumors can be excluded based on the mesothelial immunophenotype (CK pan+, calretinin+, D2-40+, WT-1+) and the absence of an identifiable primary tumor. Ancillary molecular testing further supported the diagnosis: BAP1 loss is a highly specific but poorly sensitive marker of malignant mesothelioma, and its absence (i.e., the retained BAP1 expression in the present case) does not exclude the diagnosis (4, 17). Beyond the entities discussed above, testicular and paratesticular masses with atypical morphological features warrant a broader differential diagnosis, as accurate diagnosis often requires correlating histomorphological findings with a comprehensive immunohistochemical panel. The comparatively rare sex cord–stromal tumors are a pertinent example. Leydig cell and Sertoli cell tumors, which constitute most testicular sex cord–stromal tumors, should be considered when evaluating mixed or collision tumors, and their overlapping histological features necessitate a systematic immunohistochemical approach for accurate characterization. Pepe et al. recently reported the first documented testicular Leydig–Sertoli collision sex cord–stromal tumor, underscoring that thorough morphological examination of the entire lesion combined with an appropriate immunohistochemical panel is essential for diagnosis. Key discriminatory markers include inhibin and calretinin (typically nuclear in the Leydig cell component), β-catenin and SOX9 (characteristically positive in the Sertoli cell component), and SF1 (positive in both). By contrast, mesothelioma displays a distinct immunophenotype with diffuse expression of mesothelial markers (calretinin, WT-1, and D2-40) (18).

### 3.4 The role of FAPI PET/CT in MMTVT

Fibroblast activation protein inhibitor (FAPI) PET/CT targets fibroblast activation protein (FAP) expressed by cancer-associated fibroblasts. Because mesothelioma has a prominent, fibroblast-rich stroma, FAPI imaging is mechanistically well suited to this tumor: several studies have reported that FAPI PET/CT performs comparably to, or better than, ^18^F-FDG in tumor delineation, nodal and distant staging, and prognostic assessment in pleural and peritoneal mesothelioma (19, 20). To date, however, no validated comparative data exist for FAPI imaging in mesothelioma of the tunica vaginalis or paratesticular region, and reports on this rare entity are lacking (21). In the present case, FAPI PET/CT was applied in an exploratory manner, primarily to screen for potential distant metastases exhibiting increased FAPI uptake, thereby supporting the appropriateness of radical surgery. However, several important limitations must be acknowledged. First, the scan was performed approximately three weeks after the tunica vaginalis resection. Because FAP is also expressed by activated fibroblasts during wound healing, granulation tissue formation, postoperative fibrosis, and inflammation, a recent surgical site is a well-recognized source of false-positive FAPI uptake (22, 23). In this case, the relatively ow SUV max (3.2) overlaps with the range reported for benign and reparative lesions and therefore cannot by itself distinguish viable tumor from postoperative changes (23). Because the subsequent orchiectomy specimen confirmed residual epithelioid mesothelioma, this FAPI signal most likely reflected the combined effect of residual viable tumor and superimposed postoperative reparative fibroblast activation—components that FAPI PET/CT cannot reliably separate in the early postoperative period. When clinically feasible, deferring imaging to 6–8 weeks or longer after surgery may reduce postoperative confounding. In view of these limitations, FAPI PET/CT should currently be regarded as exploratory in nature in paratesticular mesothelioma, and its clinical utility warrants validation in prospective studies employing standardized imaging protocols and appropriate postoperative time intervals.

### 3.5 Therapeutic strategies for MMTVT

Treatment of MMTVT depends mainly on the anatomical extent of the tumor, and surgical resection is the cornerstone of management. Radical inguinal orchiectomy is the primary treatment for localized disease. However, the durability of disease control cannot be inferred from short-term observation and requires prolonged follow-up; no such conclusion can be drawn from the present case. In contrast to simple hydrocelectomy, radical surgical excision has been shown to reduce local recurrence (24). When clinical or radiological findings suggest locoregional lymph node involvement, inguinal lymph node dissection may be considered (25). However, even when imaging findings are negative, clinicians remain aware of the possibility of occult metastasis (25). The importance of achieving negative surgical margins is supported by several studies. For example, a case report by Trenti et al. (26) reported long-term survival with surgery alone, without adjuvant therapy. In patients with advanced or recurrent disease, the role of adjuvant or systemic therapy remains poorly defined, owing to the extreme rarity of MMTVT and the absence of randomized controlled trials or large-scale cohort studies (5). Most treatment recommendations are extrapolated from experience with pleural mesothelioma, and the applicability of such extrapolation to MMTVT has not been systematically validated. In published case series, only a minority of patients received adjuvant therapy (radiotherapy, chemotherapy, or a combination thereof) after surgery, and the available evidence has not demonstrated a clear survival benefit (5, 21). Systemic chemotherapy regimens used in MMTVT are largely adapted from those for pleural mesothelioma. Cisplatin combined with pemetrexed, the standard first-line regimen for pleural mesothelioma, has been administered in selected MMTVT cases; however, its efficacy in this specific entity remains unclear and has not shown a definite survival benefit in the limited reports available (9, 25). Emerging immunotherapeutic and targeted agents—such as combined PD-1/CTLA-4 inhibition (nivolumab plus ipilimumab) and anti-VEGF therapy (bevacizumab)—have demonstrated efficacy in pleural mesothelioma; their value in MMTVT (27), however, remains uncertain. Case reports describing the use of immunotherapy in MMTVT have yielded inconsistent outcomes, but these remain anecdotal and insufficient to establish efficacy. In summary, the roles of adjuvant chemotherapy, radiotherapy, targeted therapy, and immunotherapy in MMTVT remain undefined; any decision to employ these modalities should be individualized.

### 3.6 Prognosis for MMTVT

The overall prognosis of MMTVT is generally unfavorable and warrants cautious clinical assessment. Overall survival is closely associated with the presence or absence of metastasis. Reported cases of long-term survival, particularly among patients treated with surgery alone, highlight several favorable prognostic factors: small tumor size (diameter <4 cm), epithelioid histology, complete surgical resection, and absence of detectable metastasis. Conversely, a cohort study by Grogg et al. of 84 patients with metastatic disease reported a median overall survival of only 18 months (8). Early detection and prompt treatment therefore remain clinically important.

### 3.7 Limitations

We acknowledge that the brief two-month follow-up in this report is insufficient to draw definitive conclusions about long-term oncological outcomes. Because MMTVT has a high propensity for delayed recurrence, the patient has been enrolled in an intensive postoperative surveillance program. This program includes contrast-enhanced computed tomography (CT) of the chest, abdomen, and pelvis every two to three months and PET/CT when clinically indicated. In addition, the ^68^Ga-FAPI PET/CT was performed only 3 weeks after the initial hydrocelectomy; because activated fibroblasts in postoperative wound healing also express FAP, the mild scrotal FAPI uptake (SUV max 3.2) likely reflected both residual tumor and postoperative reparative change and should be interpreted with caution.

## 4 Conclusion

A MMTVT is a rare but aggressive malignancy. As shown in the present case, maintaining a high index of suspicion in patients with hydrocele is important. Because its clinical and radiological features are nonspecific, a definitive diagnosis depends on histopathological evaluation. Prompt diagnosis and full assessment of metastatic status are therefore essential in all suspected cases. Preoperative FAPI PET/CT may be used to screen for lymph node and distant metastasis. Surgical resection remains the mainstay of treatment. When lymph node metastasis is present, postoperative adjuvant chemotherapy combined with radiotherapy may be considered; however, these modalities have not consistently shown a clear survival benefit. Their efficacy and safety require validation in future prospective studies. As the present follow-up is limited to 2 months, no inference regarding long-term oncological control or recurrence can be made, and extended surveillance is essential.

## Data Availability

The original contributions presented in the study are included in the article/supplementary material. Further inquiries can be directed to the corresponding author.
